# Rifle Shooting for Athletes With Vision Impairment: Does One Class Fit All?

**DOI:** 10.3389/fpsyg.2019.01727

**Published:** 2019-07-31

**Authors:** Peter M. Allen, Keziah Latham, Rianne H. J. C. Ravensbergen, Joy Myint, David L. Mann

**Affiliations:** ^1^Department of Vision and Hearing Sciences, Anglia Ruskin University, Cambridge, United Kingdom; ^2^Vision and Eye Research Unit, Anglia Ruskin University, Cambridge, United Kingdom; ^3^Department of Human Movement Sciences, IPC Research and Development Centre for the Classification of Athletes with Vision Impairment, Amsterdam Movement Sciences, Institute of Brain and Behavior, Vrije Universiteit Amsterdam, Amsterdam, Netherlands; ^4^Department of Clinical and Pharmaceutical Sciences, Life and Medical Sciences, University of Hertfordshire, Hatfield, United Kingdom

**Keywords:** visual acuity, contrast sensitivity, vision impairment, shooting, classification

## Abstract

Revised evidence-based classification criteria introduced for shooting for athletes with vision impairment (VI shooting) suggest that athletes with impaired contrast sensitivity (CS) and visual acuity (VA) should be eligible for inclusion in the sport but should all eligible athletes compete against each other in the same “class” or is more than one class necessary? Twenty-five elite VI shooting athletes took part in the study. Two measures of visual function were assessed under standardized conditions: VA (using an ETDRS logMAR letter chart, and/or a BRVT chart) and CS (using both a Pelli-Robson chart and a Mars number chart). Shooting performance, in both prone and standing events, was measured during an international VI shooting competition. Fourteen of the 25 athletes had measurable VA, and for CS, 8 athletes had measurable function with the Pelli-Robson chart and 13 with the Mars chart. The remaining athletes had function not numerically measurable by the charts and were considered to have no residual vision. There was no indication that shooting performance varied with visual function, and individuals that had residual vision had no advantage over those without vision for either prone or standing shooting. The modifications made to VI shooting, including the use of auditory tones to guide the gun barrel, appear to have successfully rendered the sport equitable for all eligible athletes. Only one class is necessary for athletes. An improved method of measuring CS in athletes with profound VI would be advantageous.

## Introduction

In order to provide structure and ensure a legitimate and equitable competitive environment in Paralympic sports, all eligible athletes undergo *classification* to be grouped into classes so that they compete against others with a similar level of impairment (International Paralympic Committee, 2007, 2017a,b). A classification system should “describe appropriately the types and severity of impairments and also consider their functional effects” (Tweedy and Vanlandewijck, 2011). The initial stage of classification is to decide whether an athlete is eligible to compete in the Paralympic sport. This is determined by the *minimum impairment criteria* (MIC), defined as the least severe impairment that limits performance in that sport (Allen et al., 2016, 2018). It is important to note that the MIC is the level of impairment that has an impact on sports performance in the unadapted rather than the adapted form of the sport and is therefore based on data collected from competitors competing in the unadapted form of the sport (Allen et al., 2018). If deemed eligible to compete then an athlete should be placed into a class according to the degree of sport-specific activity limitation caused by the impairment (Myint et al., 2016) to ensure that athletes compete against other athletes with equivalent activity limitations in the adapted form of the sport (Mann and Ravensbergen, 2018). By minimizing the differences in limitations between athletes, classification helps to legitimize competition and promote participation in para-sports.

Currently, classification for athletes with vision impairment (VI) is almost always not sport specific because the visual demands that may be required for any particular sport are not considered in most classification systems. The current classification system consists of the measurement of two elements of visual function: visual acuity (VA) and visual fields (VF) and is used by almost all sports that cater for athletes with VI. Initially, there is a medical examination to identify the underlying medical condition that has resulted in the reduction in vision. Then, all athletes are examined and, if meeting the MIC, are placed into one of three classes following classification (B3, B2, or B1, from the lowest to highest level of impairment, see Table 1). These classes are based on the definitions of low vision and blindness outlined by the World Health Organization (2004). However, because those classes were arbitrarily applied to sports, it remains uncertain whether “these classes reliably represent categories of impairment that have different effects on sport performance” (Allen et al., 2018).

**Table 1:** The criteria for the three sports classes for athletes with vision impairment.

Class	Criteria
B3	VA is between 1.0 and 1.5 logMAR (inclusive) and/or the VF is constricted to a radius of less than 20°
B2	VA is between 1.5 and 2.6 logMAR (inclusive) and/or the VF is constricted to a radius of less than 5°
B1	VA is worse than 2.6 logMAR

The Athlete Classification Code of the International Paralympic Committee (IPC; International Paralympic Committee, 2017a,b) explicitly details the need for the “development and implementation of robust classification systems that are evidence-based and sport-specific”. Although this process has been proceeding for athletes with physical or intellectual impairments, the changes to classification are only beginning to be trialed and implemented for athletes with VI (Ravensbergen et al., 2016). Our research group initiated this process for VI shooting by examining the number of classes required when considering all athletes that meet the current MIC. Shooting is a sport of special interest to athletes with VI because competitors primarily use sound rather than vision to aim the gun barrel toward the target. The air rifle is electronic and fitted with an acoustic mechanism whereby the tone becomes higher in pitch the closer to the center of the target the athlete aims. This mechanism is mounted on the air rifle, with the athlete listening to the signal through headphones directly connected to the device. An opto-electronic scoring system is used to measure the accuracy of the shots. These adaptations to the sport make it highly accessible and attractive to persons with all levels of vision impairment (Myint et al., 2016).

Myint et al. (2016) investigated the associations between shooting performance and three measures of visual function thought important for shooting (visual acuity, visual field, and contrast sensitivity) on 10 VI athletes classified according to the criteria in Table 1. In that study, athletes with VI competed using the adapted form of the sport, that is, with the assistance of the auditory guidance, because those would be the conditions that they would typically compete against each other in VI shooting. That study showed that individuals with some residual vision had no advantage over those without any light perception in the adapted form of the sport, suggesting that only one sport class was necessary for VI shooting. It appeared as though those athletes with some residual vision had no advantage over those who did not, either because residual vision is not helpful in shooting or more likely because the auditory guidance in the adapted form of the sport helps to compensate for vision loss.

Allen et al. (2016, 2018) continued this work investigating what the MIC should be for VI shooting. This work was conducted in the unadapted form of the sport, that is, in the absence of auditory guidance. The reason for this is that para-sports in most cases cater for people who have an activity limitation in the regular (i.e., unadapted) form of the sport. “An evidence-based minimum impairment criterion should ensure that only those athletes who are disadvantaged as a result of their impairment in the unadapted form of the sport are eligible to compete in the adapted form of the sport and will ensure that they compete only against others who have an impairment that does impact performance” (Allen et al., 2016). In the studies examining the MIC (Allen et al., 2016, 2018), elite able-sighted athletes shot both under standard conditions with their habitual vision and also with their vision impaired by a series of different simulation spectacles and refractive lenses. Simulation spectacles reduced both VA and contrast sensitivity (CS), while refractive lenses reduced VA with less effect on CS. A cut-off for when shooting performance was “below expected” in the presence of vision impairment was determined using habitual shooting scores. Using logistic regression and decision tree analyses, it was shown that the loss of CS, rather than VA, better predicted shooting performance. This would indicate that CS should be included in any classification system for VI athletes hoping to compete in VI shooting. Furthermore, we tentatively suggested cut-offs of approximately 0.6 logMAR for VA and approximately 1.3 logCS for contrast sensitivity. It is interesting to note that in a study investigating the effects of visual acuity on target discrimination and marksmanship, performance was significantly reduced when VA was equal or worse than 0.7 logMAR (Hatch et al., 2009).

A consequence of the new more inclusive cut-off criteria for shooting is that the original study by Myint et al. (2016) included only those athletes who met the previous inclusion criteria and so it remains unclear whether there is an advantage for those who meet the new but not the old criteria, primarily those with VA between 0.6 and 1.0 logMAR, when competing against other eligible athletes. It remains possible that their relatively better vision may provide them with an advantage over other eligible athletes, in which case a separate class would be required for them during competition. Accordingly, we sought to replicate the Myint et al. (2016) study using an increased sample size and including those athletes whose VA lies between 0.6 and 1.0 logMAR.

Another controversial issue within Paralympic sport is whether the classification system should take into account the age at which impairment was acquired. In the case of athletes with VI, there is belief that those with an acquired impairment may have an advantage in some sports because they may have had the benefit of learning key motor skills with the benefit of vision, whereas those with a congenital impairment might not (Ravensbergen et al., 2016; Tweedy et al., 2016; Mann and Ravensbergen, 2018). This effect is thought to be particularly evident in sports that involve complex motor actions for which it may be beneficial to learn with the assistance of vision. In contrast, it is conceivable that those with congenital impairment may have an advantage in sports that rely on other sensory information such as sound or touch. Some blind individuals are known to adopt “sensory substitution,” where the sensitivity of their other senses is enhanced in the absence of vision. Given that performance in VI shooting relies heavily on audition, superior audition in congenitally blind individuals could conceivable provide an advantage in VI shooting.

The aim of this study was to determine whether a significant relationship exists between vision and performance in VI shooting. Twenty-five elite VI shooters (including six whose VA was between 0.6 and 1.0 logMAR) took part in a Grand Prix competition and their shooting performance scores were correlated with measures of VA and CS. Moreover, we examined the developmental history of their impairment to establish whether the relationship was moderated by the age at which the impairment was acquired. The findings will help to determine whether separate classes are required for VI shooting if a new more inclusive VA MIC is incorporated.

## Materials and Methods

### Participants

Twenty-five (16 male/9 female) elite athletes in the sport of VI shooting took part in the study. The participants had a mean age of 49 years (SD = 11.6 years and range 15–66 years). All were competing in an international event in Innsbruck. Participation in the study was voluntary; however, all athletes attending the event agreed to participate in the project without remuneration or any other incentive. Given that VI shooting is not yet on the Paralympic program, our sample represented a considerable proportion of the eligible shooters competing on the international stage. The Faculty Research Ethics Panel at the Anglia Ruskin University, Cambridge, UK, gave ethical approval for the study. All adult participants provided written informed consent, and written informed parental consent was obtained for the 15-year-old participant. The research was conducted in accordance with the tenets of the Declaration of Helsinki.

### Procedure

#### Ocular Pathology Details

Information of participants’ ocular pathology(ies) and age of onset of ocular pathology were obtained before the measurement of visual function. The cause of participants’ vision loss was varied and was self-reported as glaucoma by five participants, retinitis pigmentosa by five, macular dystrophies by four, and trauma by three. The remaining eight reported causes of loss including optic atrophy, hydrocephalgia, and diabetic retinopathy. Five participants’ vision loss was considered congenital (age of onset <6 years) and 20 were considered to have acquired vision loss (age of onset ≥6 years).

#### Visual Function

For each athlete, two tests of visual function were performed under standardized conditions (light level 200 lux). Visual function was tested monocularly using the eye used for shooting.

VA represents the ability to recognize high-contrast characters that vary in size. Distance visual acuity (DVA) was measured using a handheld ETDRS LogMAR letter chart at 4 m (2000 Series Revised, Precision Vision, La Salle, IL, USA), with the viewing distance halved to 2 m and, if necessary, 1 m if the participant could not read the largest characters presented on the chart. Letter by letter scoring was used to record the acuity measured in logMAR units. Although a tumbling E logMAR chart is used currently for the purposes of classification, the ETDRS logMAR chart produces very similar levels of acuity (Bourne et al., 2003). If the visual acuity was too poor to be recorded using the standard letter chart (VA > 1.60 LogMAR), the Berkeley Rudimentary Vision Test (BRVT) (Precision Vision, La Salle, IL, USA) was used (Bailey et al., 2012). If the athlete could not resolve the largest test size at the closest test distance (logMAR 2.60) then a standard test of light perception was performed. For the purposes of analysis, athletes with perception of light (PL) were assigned a logMAR score of 3.0, and 4.0 logMAR was assigned to those with no perception of light (NPL) (Myint et al., 2016). A healthy young adult *without* VI would be expected to score around 0.00 logMAR.

Contrast threshold is the smallest difference in luminance that an observer can detect.

CS is the reciprocal of contrast threshold, and higher logCS scores indicate better CS. Two test methods were used to assess CS, the Pelli-Robson chart (Haag-Streit UK, Essex, UK) (Pelli et al., 1988), and the Mars chart (Mars Perceptrix, Chappaqua, NY, USA) (Arditi, 2005; Dougherty et al., 2005).

1. The Pelli-Robson chart comprises eight rows each showing two triplets of letters, therefore, showing 16 triplets in total. A test distance of 1 m was used. Each triplet is of equal contrast, starting with a contrast of 0.00 logCS, and each successive triplet decreasing by 0.15 logCS, with the final triplet being 2.25 logCS. Athletes were asked to read each letter from left to right, top to bottom commencing with the 0.00 logCS triplet, with the test stopping when two letters from a triplet were incorrectly named. The Elliot method of scoring was used, where each correctly named letter was scored as 0.05 logCS (Elliott et al., 1990). The expected score on the Pelli-Robson test for young adults *without* VI is 1.70 ± 0.08 logCS units (Dougherty et al., 2005).
2. The Mars number chart consists of eight rows of six numbers. The test commenced at 50 cm but consistent with the test instructions for the Mars test (Mars Perceptrix, 2010), participants were allowed to change their working distance to minimize the chance that VA limited test performance. LogCS scores on the Mars chart range from 0.00 to 1.92 logCS with each successive number lower in contrast by 0.04 logCS units. Participants were asked to read out the numbers, with the test stopping when two consecutive numbers were incorrectly named. The final CS was the contrast level of the final correct number minus 0.04logCS for every incorrectly named number. The expected score on the Mars test for young adults *without* VI is 1.72 ± 0.06 logCS units (Dougherty et al., 2005).

#### Shooting Performance

There are two different 10 m air rifle competition events for VI shooting: standing and prone events. In the standing position, the athlete supports the weight of the rifle while shooting, whereas in the prone competition, the athlete sits on a stool and rests their arm and rifle on a table (<90 cm diameter). Sighted assistants are permitted to aid the VI athlete in their set up and general positioning but not with the actual shot. According to the rules of the International Blind Sports Federation^1^, competition takes place across two rounds, a qualifying and final round, with men and women competing in the same competition against each other. In the qualifying round, athletes shoot 60 times at a target of 10 concentric rings. Scoring is such that the athlete scores 10 for a hit in the central ring, nine for the next, and so on. The 10 rings are then subdivided into 10 score zones, each representing an increment of 0.1 (so the highest score for an individual shot is 10.9). The eight best-scoring shooters progress to the final round. During the final, the lowest scoring athletes are progressively eliminated from the competition and the best scoring athletes remain. The cumulative scores determine the final positions; however, the nature of the elimination process means that athletes take an unequal number of shots during the final. In this study, performance was assessed, over two consecutive days, during both the prone and standing events. The primary outcome measure was the score after the qualifying round as it was this score that was available for all participants and for which each participant took an identical number of shots.

#### Statistical Methods

Data were analyzed using IBM SPSS Statistics version 24. Groups were compared using independent and repeated-measures *t*-tests as appropriate. Effect sizes are reported for significantly different comparisons using Cohen’s *d*. Correlations are presented using Kendall tau (*τ*) on the basis of the sample size available.

## Results

There was no difference in the shooting scores between males and females for either the prone [male (*n* = 16), 553.3 ± 37.7; female (*n* = 9), 564.3 ± 31.9; *t*(23) = −0.74, *p* = 0.47] or standing competitions [male (*n* = 13) 472.9 ± 55.0; female (*n* = 9) 456.9 ± 85.6; *t*(20) = 0.53, *p* = 0.60], and therefore, all athletes were considered together in all analyses.

Mean DVA in the shooting eye was 2.34 ± 1.43 logMAR (range 0.24–4.00 logMAR) for the 25 athletes. There were two participants with PL and nine with NPL: these 11 were considered to have “no residual vision.” Of the 14 subjects with residual measurable VA, mean VA was 1.18 ± 0.60 logMAR, and there were six participants with VA better than 1.0 logMAR.

Contrast sensitivity showed a strong floor effect, with only some of the 25 athletes having measurable (>0.00 logCS) contrast sensitivity or “residual vision” on each chart (13 for the Mars chart and 8 for the Pelli-Robson chart). Mean CS for all observers on each chart was 0.34 ± 0.44 logCS (range 0–1.16 logCS) for the Mars and 0.24 ± 0.43 logCS (range 0–1.20 logCS) for the Pelli-Robson. Mars and Pelli Robson contrast sensitivity scores were highly correlated (Kendall *τ* 0.73, *p* < 0.001), but with Pelli Robson scores (mean 0.24 ± 0.43 logCS) being significantly lower [*t*(24) = −2.61, *p* < 0.05, Cohen’s *d* = 0.23] than the Mars scores (0.34 ± 0.43 logCS).

All 25 athletes took part in the prone competition. Three athletes did not take part in the standing competition: these athletes had VA/CS in the shooting eye of 4.0 logMAR/0.0 logCS, 3.0 logMAR/0.0 logCS, 0.90 logMAR/0.35 logCS. There were therefore 22 athletes with scores for both standing and prone competitions, with their scores for prone (563 ± 33) being significantly higher [*t*(21) = −8.2, *p* < 0.001, Cohen’s *d* = 1.81] than for standing (466 ± 68). Consideration of the requirement for different classes is therefore presented for prone and standing conditions separately.

### Prone

**Figure 1:**
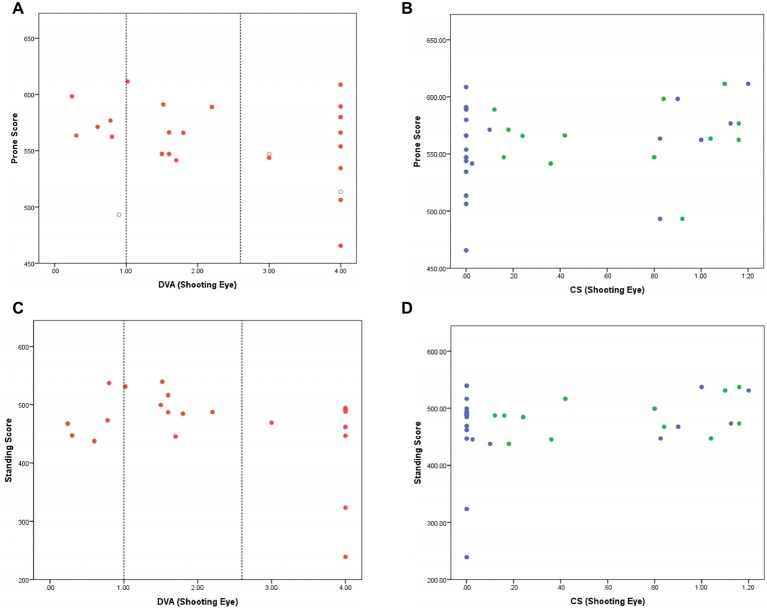
**(A–D)** Shooting score in the prone **(A,B)** and standing **(C,D)** competitions are shown as a function of distance VA (**A** and **C**; red) and CS (**B** and **D**; green for Pelli Robson test and blue for Mars test) of the shooting eye. Closed symbols indicate athletes who shot in both prone and standing competitions. Open symbols indicate athletes who shot in the prone competition only. In the VA figures **(A,C)**, vertical lines indicating 1.0 logMAR (current minimum entry criterion to VI shooting) and 2.6 logMAR [poorest vision measureable with the BRVT; above this level athletes have PL (assigned 3 logMAR) or NPL (assigned 4 logMAR)] are shown. Young adults without VI typically score 0.00 logMAR. In the CS figures **(B,D)**, participants with no measurable function have a score of 0.00 logCS. Young adults without VI typically score 1.70 logCS units.

There was no indication that shooting scores in the prone competition depended on distance VA (Figure 1A; Kendall *τ* correlation −0.22, *p* = 0.14). Although the lowest score was achieved by an athlete with NPL, shooting scores for the athletes with NPL were registered across the range of different performance scores, including an athlete with the second highest score. Newly included athletes with VA 0.6–1.0 logMAR performed well but not markedly better than what others with less vision were capable of scoring.

Shooting performance was compared between those with (logCS>0) and without measurable contrast sensitivity (logCS = 0) on each chart because of the high number of athletes with unmeasurable function on each chart. There was no significant difference in prone shooting performance between these groups for either of the CS charts [Pelli Robson: *t*(23) = 0.72, *p* = 0.48; Mars: *t*(23) = 1.00, *p* = 0.33].

### Standing

Similar to the prone competition, there was no apparent relationship between scores in the standing competition and DVA (Figure 1C; Kendall *τ* correlation −0.11, *p* = 0.49). There was no significant difference in standing shooting performance between groups for either of the CS charts [Pelli Robson: *t*(20) 0.50, *p* = 0.62; Mars: *t*(20) 1.41, *p* = 0.17]. Two athletes with NPL did record the lowest scores, but the remainder of the athletes with NPL shot competitively when compared with those with better levels of VA and CS.

### Differences Between Prone and Standing Scores

Across the 22 participants who shot in both the prone and standing competitions, prone scores were always higher than standing scores, with the mean difference in scores between the two competitions being 96 ± 55 points. The difference in scores between the two protocols was compared for athletes with no residual vision (PL or NPL; *n* = 8; mean difference between prone and standing scores = 121 ± 78) and athletes with measurable VA (*n* = 14; mean difference between prone and standing scores = 82 ± 33). The difference in scores between prone and standing competitions did not depend on whether the athlete had residual vision or not [*t*(20) = 1.64, *p* = 0.12].

### Differences Between Athletes With a Congenital Versus an Acquired Vision Loss

There was no significant difference in the performance of those with congenital visual loss (age of onset <6 years) and those with acquired visual loss (age of onset ≥6 years) in either the prone [congenital (*n* = 5) 569 ± 27, acquired (*n* = 20) 554 ± 37; *t*(23) 0.83, *p* = 0.42] or standing [congenital (*n* = 5) 485 ± 21, acquired (*n* = 17) 461 ± 77; *t*(20) 0.70, *p* = 0.49] scores.

## Discussion

The aim of this study was to determine whether a significant relationship exists between visual function and performance in VI shooting. We have previously shown that CS and to a lesser extent VA are important visual function measures and should be used for the classification of athletes hoping to compete in VI shooting (Allen et al., 2016, 2018). In this study, we wanted to judge performance in the adapted form of the sport (with auditory guidance etc.) under the newly proposed minimum inclusion criteria and hence measured and analyzed measures of visual function including distance VA and contrast sensitivity of the shooting eye when participants competed in the prone and standing adapted forms of VI shooting. We have previously demonstrated that if athletes are classified using the current VA cut-off of 1.0 logMAR then those with better VA and/or CS did not outperform those with worse VA/CS. In fact, superior shooting performance was sometimes achieved by the athletes with poorer vision (Myint et al., 2016). The motivation behind the current study was to repeat our previous work with a larger sample size and to include participants whose VA fell between 0.6 and 1.0 logMAR. These athletes would previously have been classified as ineligible to compete in VI shooting based on their VA but will be eligible to compete if the MIC changes to 0.6 logMAR.

Figures 1A–D show that shooting performance in both the standing and prone competitions is not significantly influenced by either VA or CS. It is important to note that the five athletes with DVA better than 1.0 logMAR (dotted vertical line in Figures 1A,C) who would not be included in VI shooting under the existing criteria performed similarly to those with worse VA, suggesting that they do not hold a performance advantage over those with less visual function. Moreover, there was no difference in the scores of males and females in either the prone or standing competitions.

We were interested in establishing whether sensory substitution in congenitally blind individuals might lead to a performance advantage in VI shooting (Kupers and Ptito, 2013; Ravensbergen et al., 2016). However, there were no significant differences in shooting performance in either the standing or prone events when comparing athletes with a congenital and an acquired impairment. Therefore, it appears, on the basis of the evidence available, that individuals with a congenital impairment do not possess an advantage in VI shooting. This is an issue worthy of further consideration as the sport begins to grow, and more athletes are attracted to the sport given the limited number of individuals currently competing at the international level.

The range of performance achieved by those with NPL (4.0 logMAR) is noteworthy. While some of these athletes did perform poorly, others with NPL performed at a level similar to that achieved by others who did have measurable vision. Factors that best predict performance in unadapted shooting are related to the athlete’s ability to maintain concentration and control anxiety (Vickers, 2001; Janelle, 2002) and the influence of those factors on aiming accuracy, stability of hold, cleanness of triggering, and timing of triggering (Ihalainen et al., 2016). These factors may also be dependent on the athlete’s ability to maintain balance (Vickers and Williams, 2007; Sattlecker et al., 2014). Prone scores were higher than standing. This might demonstrate the additional postural stability required for the standing event, especially considering postural stability is reduced in people with vision loss (Bouchard and Tetreault, 2000; Portfors-Yeomans and Riach, 2008; Willis et al., 2013) but can be improved with exercise (Campbell et al., 2005; Chen et al., 2012). Future research to investigate the factors influencing performance of those with NPL would be valuable and could include aspects indirectly related to vision, such as postural stability.

A limitation of previous work (Myint et al., 2016) is that a Pelli-Robson chart was used to measure CS. The level of VA required to perform the Pelli-Robson test resulted in a measurable value not being possible for many participants. If CS is to be included in a classification test, battery for VI sport then a method of measuring CS in athletes with moderate to severe visual loss is required. In the current study, CS was able to be measured in only 8 of 25 participants when using the Pelli-Robson test and in 13 of the 25 participants using the Mars test. From these data, we would advocate the use of the Mars test for the classification of athletes with vision impairment in VI shooting. However, a method that increases the number of athletes able to achieve a measurable result with the test is desirable.

The performance data reported in this study were collected at a Grand Prix competition for VI shooters. Although this is strength of the study because the athletes were tested in a highly representative environment and would have been motivated to perform to the best of their ability, it could also be considered a weakness because the athletes were likely to be experiencing anxiety while competing in this important event. In future studies, a measure of anxiety such as the Mental Readiness Form (Krane, 1994) could help in the interpretation of performance during an important event. Another potential limitation of the study is that the measurement of visual acuity and contrast sensitivity relies on the subjective and therefore honest responses of participants. These subjective tests are susceptible to intentional misrepresentation, where a potential athlete may willfully perform poorly to appear as though their vision is worse than it actually is (Ravensbergen et al., 2018; Krabben et al., 2019). A more objective method of measuring visual acuity and contrast sensitivity is desirable because it would reduce the risk of intentional misrepresentation.

The modifications made to the VI shooting would appear to successfully render the sport equitable for athletes of both sexes with VA of less than or equal to 0.6 logMAR. Therefore, as there is no evidence to support the need for more than one class, we recommend all athletes to compete in a single class once they are deemed eligible to compete in VI shooting.

## Data Availability

The datasets generated for this study are available on request to the corresponding author.

## Ethics Statement

The Faculty Research Ethics Panel at Anglia Ruskin University, Cambridge, UK, gave ethical approval for the study. All subjects provided informed consent and the research was conducted in accordance with the tenets of the Declaration of Helsinki.

## Author Contributions

All authors conceived and designed the study. PA and JM collected the data, PA and KL analyzed the data. All authors wrote, reviewed, edited, and approved the manuscript.

### Conflict of Interest Statement

The authors declare that the research was conducted in the absence of any commercial or financial relationships that could be construed as a potential conflict of interest.

## Footnotes

^1^http://www.ibsasport.org/sports/files/16-Rules-IBSA-Shooting-Rulebook-2011-2013.pdf
